# Plants and Fevers

**Published:** 1902-03-29

**Authors:** 


### Editor's Letter-Box.

[Our Correspondents are reminded that prolixity is a great bar to publication, and that brevity of style and conciseness of statement greatly facilitate early insertion.]

### PLANTS AND FEVERS.

"Mr. B. Crompton" writes: In *The Garden* for February 22nd there is an illustration of moschosma riparium which seems to be the same as a plant called by the Kaffirs "Borza." There is also a description, but it omits the fact that it has a wonderful effect on some forms of fever. The colour of the flower in its native state is a decided grey or lavender. The part used is the leaf, of which a decoction is made. It might be useful (probably not alone) for the enteric at the front; the temperature goes down slowly, but seems less inclined to run up again than with some medicines. It might also be beneficial in influenza and malaria. It grows commonly near the Princess Christian Hospital, Pine Town, Natal. The best representation I know of moschosma riparia is the drawing by Miss Freda South, in "Natal Plants," of which there are copies at Kew.

